# The Role of Alpha-1 and Alpha-2 Adrenoceptors in Restraint Stress-Induced Liver Injury in Mice

**DOI:** 10.1371/journal.pone.0092125

**Published:** 2014-03-28

**Authors:** Qing Zhu, Liwei Gu, Yimei Wang, Li Jia, Zengming Zhao, Shuangqing Peng, Linsheng Lei

**Affiliations:** 1 School of Pharmaceutical Sciences, Southern Medical University, Guangzhou, China; 2 Evaluation and Research Center for Toxicology, Institute of Disease Control and Prevention, Academy of Military Medical Sciences, Beijing, China; 3 Qinghaosu (Artemisinin) Research Center, Institute of Chinese Materia Medica, China Academy of Chinese Medical Sciences, Beijing, China; University of Navarra School of Medicine and Center for Applied Medical Research (CIMA), Spain

## Abstract

Acute stress affects cellular integrity in many tissues including the liver, but its underlying mechanism is still unclear. The aim of the present study was to investigate the potential involvement of catecholamines and adrenoceptors in the regulation of acute restraint stress-induced liver injury. Restraint was achieved by placing mice in restraint tubes. Mice were treated with either an *α*-l antagonist, prazosin, an *α*-2 antagonist, yohimbine, a β-l antagonist, betaxolol, a β-2 antagonist, ICI 118551, or a central and peripheral catecholamine depleting agent, reserpine, and followed by restraint stress. Assessment of liver injury (serum alanine aminotransferase (ALT), aspartate aminotransferase (AST) , hepatic total GSH, GSSG and GSH/GSSG ratio) , histopathology and of apoptosis, by TUNEL (terminal deoxynucleotidyl transferase-mediated dUTP nick-end labelling) assay and western blotting, was performed. Three hours of restraint stress resulted in liver injury, as indexed by elevated serum transaminase levels, decreased hepatic total GSH levels and GSH/GSSG ratio, increased hepatic GSSG levels as well as enhanced hepatocytes apoptosis. Either reserpine or prazosin or yohimbine was found to attenuate liver injury. Furthermore, prazosin and yohimbine protected against restraint-induced hepatocytes apoptosis through attenuating the activation of caspases-9 and -3 and reducing the Bax/Bcl-2 ratio. These results suggest that α-1 and α-2 adrenoceptors mediate restraint-induced liver oxidative injury through caspase-9 and Bcl-2 family of apoptotic regulatory proteins.

## Introduction

Stress is an ever-present part of modern life. The general concept of “stress” describes the state of a living organism when, under the influence of internal or external stimuli or “stressors”, the dynamic equilibrium of the organism (homeostasis) is threatened [1]. Stressors can include physical or mental forces or combinations of both. The adaptive response to stressors comprises the activation of the hypothalamic–pituitary–adrenal (HPA) axis and components of the sympathoadreno-medullary (SAM) system, releasing the key peripheral mediators, i.e. glucocorticoids and catecholamines [2].

For a long time, stress had been implicated as a cofactor in the severity and progression of a number of diseases. The current focus on the stress and disease phenomenon is directed towards the interactions of the immune system, the CNS, cardiovascular disease, liver injury, HIV/AIDS and carcinomas [3], [4], [5]. Studies reveal that stress plays a role in suppressing functions of specific immunity, in triggering or worsening depression, cardiovascular disease, and liver injury,and in speeding the progression of HIV/AIDS and carcinomas [6]. Stressor-elicited endocrine response provides the key pathway linking psychological stress. Glucocorticoids, the primary effector of HPA activation, regulate a broad range of physiological processes, including anti-inflammatory responses, metabolism of carbohydrates, fats and proteins, and gluconeogenesis. Catecholamines, which are released in response to SAM activation, work in concert with the autonomic nervous system to exert regulatory effects on the cardiovascular, pulmonary, hepatic, skeletal muscle, and immune systems. Prolonged or repeated activation of the HPA and SAM systems can interfere with their control of other physiological systems, resulting in increased risk for physical and psychiatric disorders [6].

Experimental research in animals is an effective method of investigating stress, since animal models are much easier to control environmentally, and that these models are genetically identical. Animal immobilization or restraint is known to be an applicable, easy and convenient model to induce both psychological and physical stress. Immobilization or restraint can induce psychological escape reaction and physical muscle work, which result in restricted mobility and aggression [7]. In the recent 15 years, ‘‘restraint stress’’ continues to dominate stress-induced methodology, especially for experiments that employ rodents as subjects [8]. Restraint stress not only enhances xenobiotic-induced hepatotoxicity [9], but also affects cellular integrity in many tissues including heart, stomach and brain, especially liver [10], [11]. All these alterations may be caused by catecholamines. Sustained elevation of plasma norepinephrine by means of miniosmotic pump implantation in the peritoneal cavity can cause hepatocyte injury and depresses liver function [12]. In recent years, it becomes clear that liver injury, caused by stress, is the consequence of enhanced free radical generation and altered antioxidant enzyme activities [13], [14], [15].

Psychological or physical stress stimulates the adrenal medulla to secrete the two catecholamines, adrenaline (epinephrine) and noradrenaline (norepinephrine), which initiate the “fight or flight” response. Many researchers have found that catecholamins and adrenoceptors are implicated in modulation of cytotoxicity and tissues injury including liver. It has been reported that adrenaline can promote hydroxyl radical generation in isolated rat hepatocytes [16]. In a study [17] conducted on MI-induced heart failure in rats, it is observed that apoptosis is attenuated by different β adrenoceptor blocking agents. Previous data have shown that concomitant administration of propranolol with ethanol significantly attenuates the sensitizing effect of ethanol on LPS-induced liver damage [18]. Furthermore, interactions of the HPA axis and SAM occur at several levels in the periphery and the brain [19]. For example, several studies have shown that pharmacological blockade of α or β receptors can inhibit the pathological hyperfunction of HPA axis during chronic stress [20]. However, previous studies have primarily focused interest on the role of the HPA axis and glucocorticoid in the stress response.

The aim of the present study was to investigate the potential involvement of catecholamines and adrenoceptors in the regulation of acute restraint stress-induced liver injury. Animals were exposed to restraint for 3 h, an acute stress paradigm shown to induce liver injury. The role of catecholamines and adrenoceptors in the regulation of stress was assessed in mice exposed to α or β blockade and catecholamine depletion before restraint stress. After restraint session, liver injury parameters were assessed.

## Methods

Animal experiments were carried out in strict accordance with the recommendations of the Guide for the Care and Use of Laboratory Animals of the National Institutes of Health and approved by the Animal Ethics Committee of the AMMS. To minimize the suffering of laboratory animals, all pharmacological interventions were done under anaesthesia. The animal tissues for ex vivo experiments were taken post mortem, immediately after all animals were sacrificed by cervical dislocation.

### Animals

Adult male BALB/c mice (8–10 weeks old) were obtained from the Academy of Military Medical Science (Beijing, China). All animals were housed in an environmentally controlled room with a 12 h light/dark cycle and allowed free access to food and water. The mice were allowed to acclimatize themselves to the colony for 3 days before the experiments began. The temperature in the colony room was maintained at 20–26°C. The experimental protocol was approved by the Animal Ethics Committee of the Academy of Military Medical Sciences in China and followed the Guide for the Care and Use of Laboratory Animals of the National Institutes of Health.

### Drug administration

Prazosin(Sigma-Aldrich) was dissolved in saline (0.1 mg/ml) and a dose of 1 mg/kg was administered intraperitoneally 30 min before restraint stress to block *α*-1 receptors. Yohimbine (Sigma-Aldrich) was dissolved in saline (0.2 mg/ml) and a dose of 2 mg/kg was administered intraperitoneally 30 min before restraint stress to block *α*-2 receptors. Betaxolol (Santa Cruz) was dissolved in 20% dimethylsulfoxide in saline (2 mg/ml) and a dose of 20 mg/kg was administered intraperitoneally 30 min before restraint stress to block β-1 receptors. ICI 118,551 (Santa Cruz) was dissolved in saline (0.5 mg/ml) and a dose of 5 mg/kg was administered intraperitoneally 30 min before restraint stress to block β-2 receptors. Reserpine (Sigma-Aldrich) was dissolved in saline (0.2 mg/ml) and a dose of 2 mg/kg was subcutaneously injected 24 h prior to restraint stress in order to achieve a permanent depletion of catecholamines. All of doses were chosen based on previous studies [21], [22], [23], [24].

### Experimental procedure

Animals were fasted overnight before the experiments. On the day of the experiment (starting at 8:00 AM), mice were introduced into restraint tubes for varying periods. The restraint tubes were well ventilated and prevented animals from turning or ambulation, but they did not squeeze the mice. At the beginning of the restraint period, food and water were removed from control and restrained animals. After restraint, mice were immediately anesthetized with sodium pentobarbital (60 mg/kg), blood was collected from the abdominal aorta and they were killed by cervical dislocation. The liver was removed and was rinsed in saline; a same lobe of the liver was fixed in 10% phosphate-buffered formalin and embedded in paraffin for histological analyses. The remaining liver was snap-frozen in liquid nitrogen and stored at −80 °C.

### Determination of Serum enzymes

Blood samples were collected and allowed to clot overnight at 4 °C. The serum was separated by centrifugation at 3000×g for 10 min. Serum alanine transaminase (ALT) and aspartate transaminase (AST) were then determined using commercial test kits (Biosino, Beijing, China) following the manufacturer's instructions. These enzyme activities were expressed as an international unit (IU/L).

### Measurement of total hepatic GSH (GSH+GSSG), GSSG and GSH/GSSG ratio

Total soluble GSH and GSSG were measured in the liver homogenate using the enzymatic recycling method [25]. Briefly, about 100 mg of tissue was homogenized in 10 volumes of cold 5-sulfosalicylic acid (5%). Protein was precipitated and the supernatant was used to determine reduced glutathione (GSH) and glutathione disulphide (GSSG). Total glutathione (GSH + GSSG) was determined by spectrometry using 5, 5-dithiobis (2- nitrobenzene acid) (DTNB), NADPH and GSH reductase at 412 nm. GSSG determination was similar for total glutathione, except that GSH was first derivatized for 1 h with 2-vinylpyridin. Change in absorbance at 412 nm was recorded for 3 min. The GSH level was calculated by subtracting GSSG content from the total glutathione content (GSH  =  total GSH-2GSSG). Results were expressed in μmol of GSH or nmol of GSSG per g of liver.

### Histopathology and immunohistochemistry

Formalin-fixed tissue samples were embedded in paraffin and 4μ m sections were cut. Sections of liver were stained with hematoxylin and eosin (H&E) for routine histological examinations and morphometric analyses.

Apoptosis detection was performed using a TUNEL peroxidase apoptosis detection kit (Roche Molecular Biochemicals, Indianapolis, IN) according to the manufacturer's instructions. Briefly, 3μ m thick deparaffinised sections were pretreated with proteinase K (20μ g/ml) for 15 minutes at room temperature. After washing in PBS, the endogenous peroxidase was inactivated in 3% H_2_O_2_ for 10 minutes, followed by incubation with TdT enzyme at 37°C for one hour. After this incubation, sections were incubated with antidigoxigenin conjugate at room temperature for 30 minutes followed by incubation with diaminobenzidine (DAB) solution. Slides were counterstained with hematoxylin and mounted. The TUNEL-positive cells were checked by light microscopy. Apoptotic hepatocytes were identified by positive TUNEL staining and morphology (cell shrinkage, chromatin condensation and/or imagination, and formation of apoptotic bodies). One thousand cells were counted in each case by using high power microscope to establish the ratio of apoptotic cells (apoptotic index).

### Western blot analysis

Frozen liver tissue (approximately 50 mg) was homogenized in 0.5 ml lysis buffer containing ice-cold 50 mM Tris, pH 7.4, 150 mM NaCl, 5 mM EDTA, 0.1% SDS and 0.5% PMSF. The liver homogenate was centrifuged at 10,000×g for 20 min at 4°C and protein concentration of the resulting supernatant was determined by the BCA method.

Liver lysate protein (20μ g) of each sample was separated by 12% SDS-PAGE and then electrophoretically transferred onto a PVDF membrane (Amersham Biosciences). The membranes were blocked with TBS (40 mM Tris, pH 7.6, 300 mM NaCl) with 0.1% Tween 20 containing 0.5% nonfat dry milk, for 1 h at room temperature. The membranes were then incubated overnight with primary antibodies, rabbit polyclonal anti-mouse actin, caspase-3, caspase-9, Bcl-2, and Bax (Santa Cruz Biotechnology, Santa Cruz, CA) in TBST at 4°C. After five washes with TBST, membranes were incubated with horseradish peroxidase-conjugated goat anti-rabbit IgG (Zymed Laboratories Inc.) for 45 min at room temperature. Immunoreactive proteins were detected by enhanced chemiluminescence method kit (Wuhan Boster Biological Technology Ltd, Wuhan, China).

### Statistics

All results were expressed as mean±SD. Comparisons between multiple groups were performed with one-way ANOVA followed by a post hoc Bonferroni test. If the data were not normally distributed, we used the Kruskal-Wallis Test (nonparametric ANOVA) followed by Dunn's Multiple Comparisons Test. Results were considered significant when p-values were less than 0.05.

## Results

### Effect of restraint stress on serum enzyme activities, hepatic glutathione content and hepatic histopathology

**Figure 1:**
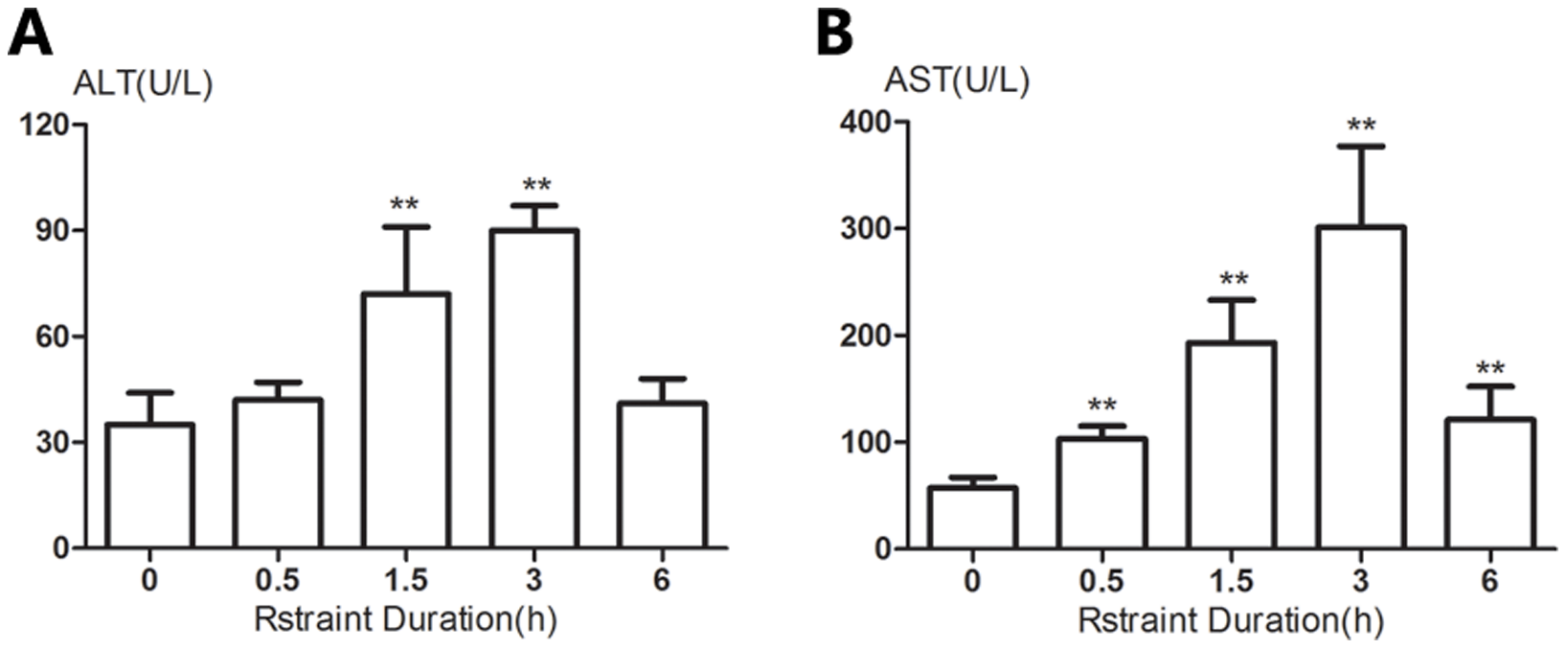
Changes in serum ALT and AST activities in mice subjected to restraint stress in varying time periods. Unrestrained animals were maintained in their cages. At indicated time points, animals were sacrificed and serum ALT (A) and AST (B) activities were determined. Results are expressed as means±SD. There are 5 animals per group. Significance of the differences *vs* unrestraint animals is given by ** *p*<0.01.

After the mice were subjected to restrain, serum ALT and AST activities were increasing over the first 3 h and then a decline at 6 h time point was observed (Fig. 1). Both ALT and AST activities at the 3 h time point reached or exceeded 3 times of the normal levels. According to “Hy's Law” adopted by the U.S. Food and Drug Administration (FDA), severe hepatocellular injury has been defined as being cases where the serum ALT or AST is > 3 times the upper limit of normal [26].

**Figure 2:**
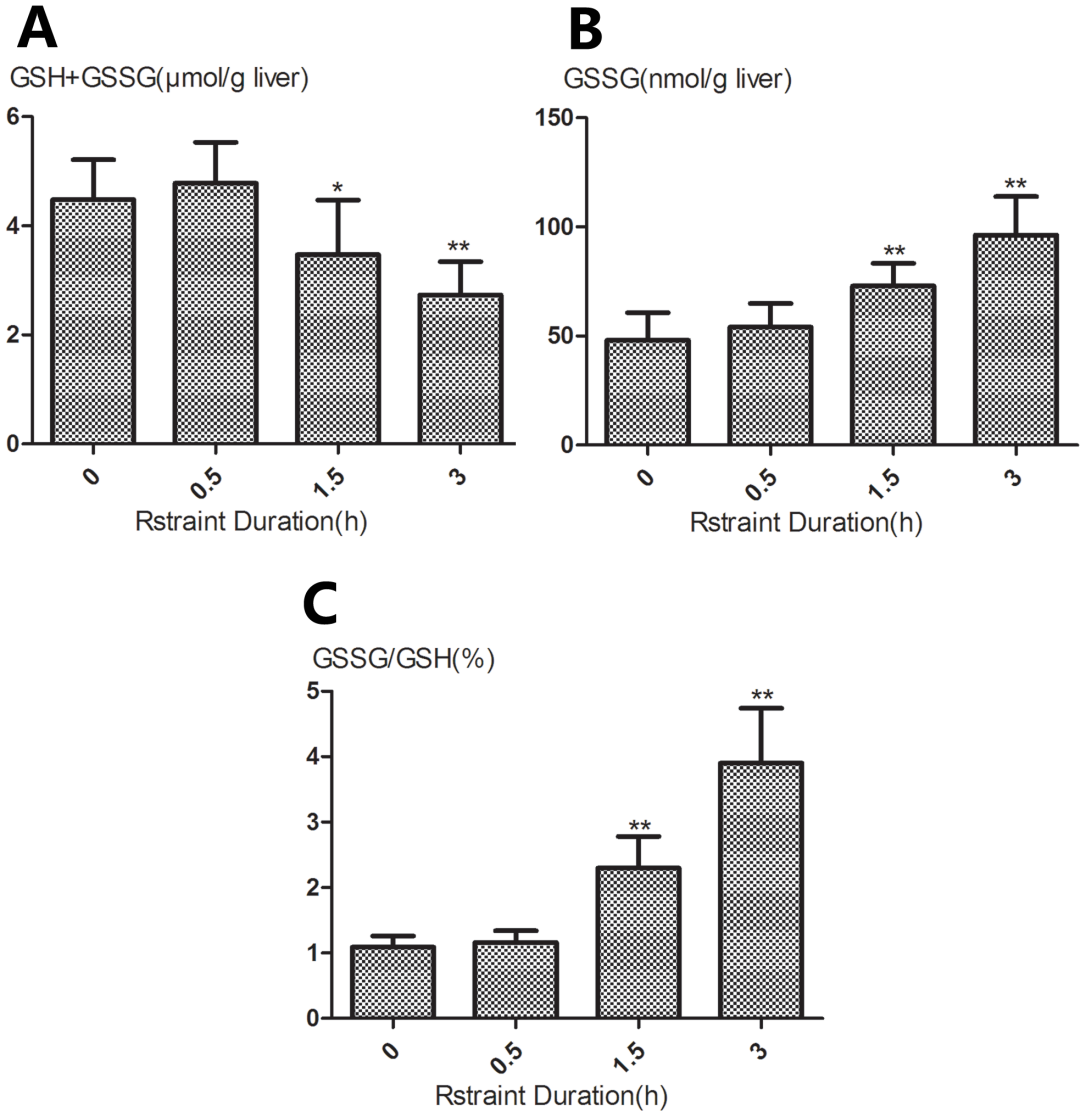
Changes in hepatic glutathione content in mice subjected to restraint stress in varying time periods. Liver content of total glutathione (GSH+GSSG) (A), GSSG (B) and the GSSG-to-GSH ratio (C) were measured at the indicated time points. Results are expressed as means±SD. There are 5 animals per group. Significance of the differences *vs* unrestraint animals is given by * *p*<0.05, ** *p*<0.01.

Similar to plasma ALT and AST activities, restrained animals exhibited a progressive changes in hepatic GSH (GSH+GSSG) (Fig. 2, A), GSSG (Fig. 2, B) and GSH/GSSG ratio (Fig. 2, C). Hepatic GSH and GSH/GSSG content were significantly higher after 1.5 and 3 h of restraint in the restrained group than those in unrestrained animals (*p*<0.05 or *p*<0.01). However, restrained animals exhibited significantly lower GSSG content after 1.5 and 3 h of restraint compared with unrestrained animals (*p*<0.01).

**Figure 3:**
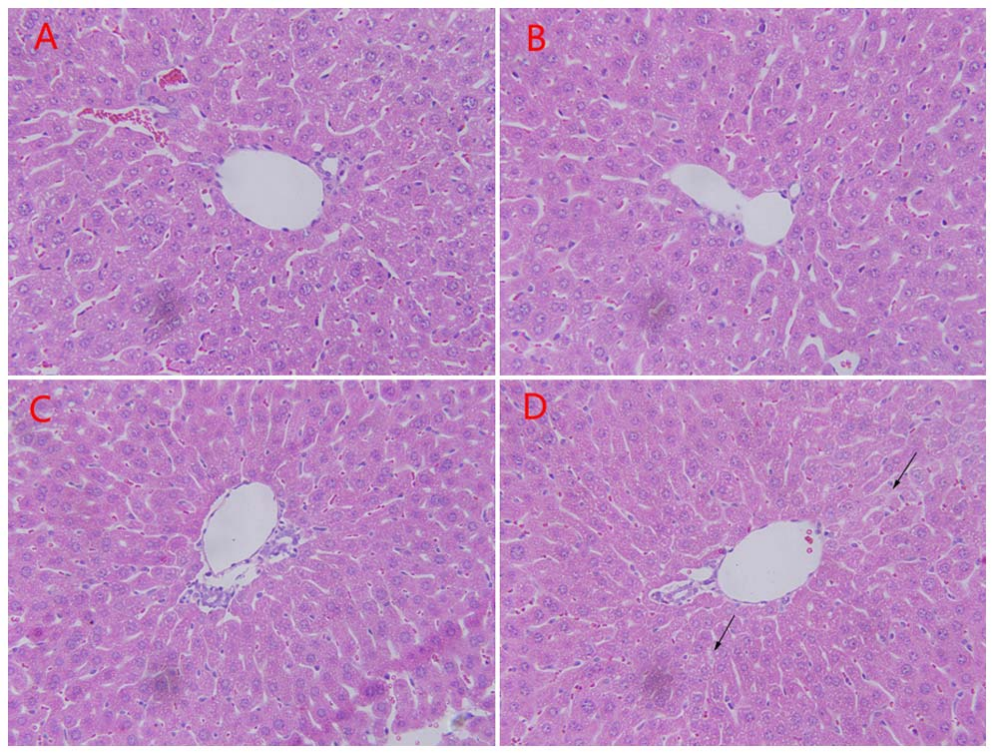
Hepatic histopathology in mice subjected to restraint stress in varying time periods. Light photomicrographs of liver sections were obtained from control mice (A), and mice exposed to 0.5 h (B), 1.5 h (C), 3 h (D) restraint stress. Most of the liver parenchyma had normal appearance either after 0.5 h or 1.5 h restraint. Only mice subjected to 3 h restraint showed slight cytoplasmic relaxation (black arrows). Magnification×400.

However, histopathological examinations did not reveal obvious liver lesion in the restraint mice compared to the control (Fig. 3). Only mice exposure to 3 h restraint showed mild hepatic degeneration with the presence of slight cytoplasmic relaxation.

### Roles of catecholamines and adrenoceptors in restraint stress-induced liver injury

**Figure 4:**
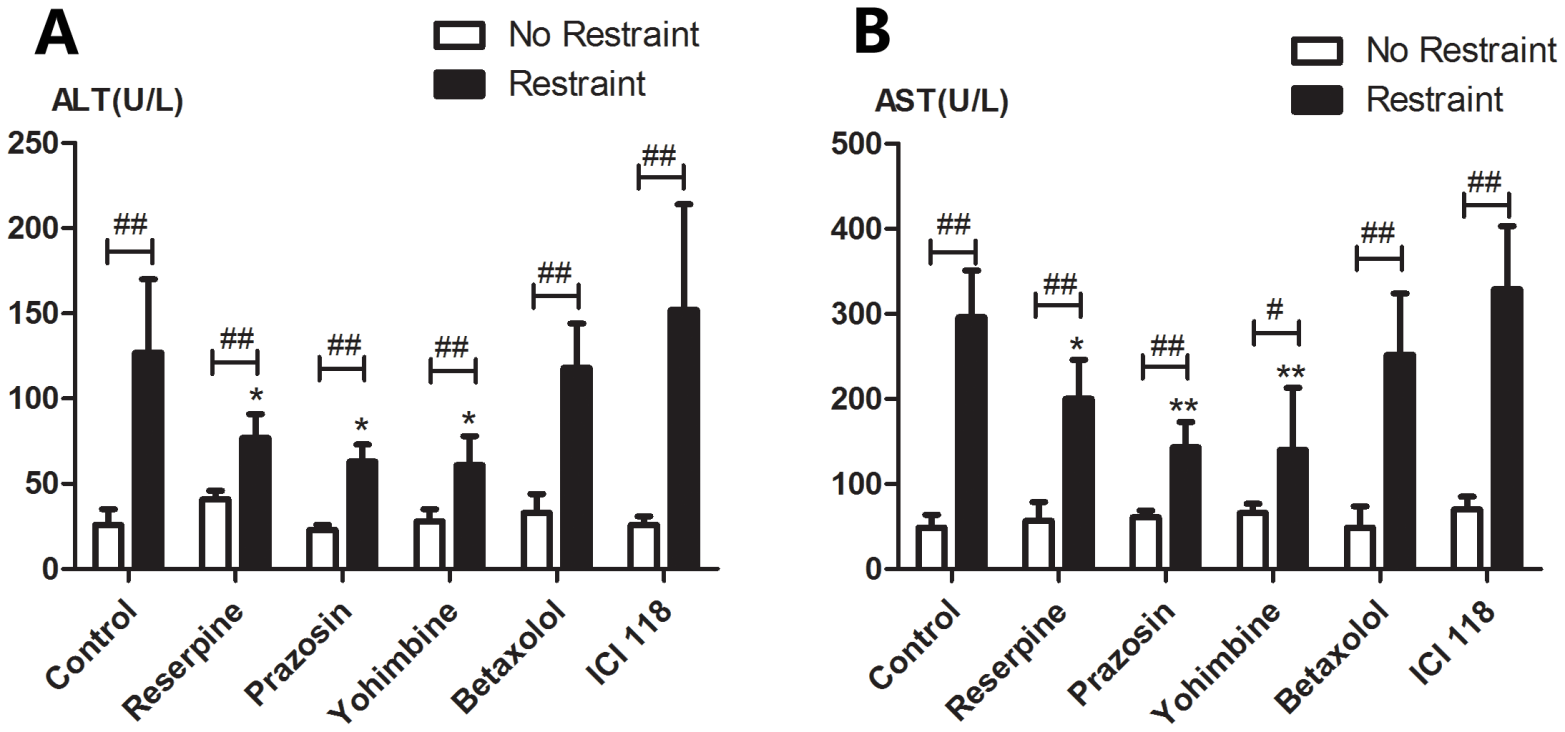
Changes in serum ALT and AST activities after pharmacologic manipulation of catecholamine levels and adrenergic receptors in mice followed by exposure to 3 h of restraint. Unrestrained animals were maintained in their cages. Mice were treated by vehicle control, reserpine (2 mg/kg), prazosin (1 mg/kg), yohimbine (2 mg/kg), betaxolol (20 mg/kg) or ICI 118,551 (5 mg/kg) before restraint. Drug-treated animals with 3 h of restraint or unrestraint were sacrificed at the same time points and serum ALT (A) and AST (B) activities were determined. Results are expressed as means±SD. There are 5 animals per group. * *p*<0.05, ** *p*<0.01 compared with control animals (untreated restraint animals); ^#^ *p*<0.05, ^##^ *p*<0.01compared with control animals (corresponding unrestrained animals).

In this experiment, we evaluated whether pharmacologic manipulation of catecholamine level and adrenergic receptors affected restraint stress-induced liver injury. Treatment with reserpine, prazosin, yohimbine, betaxolol and ICI 118551did not influence ALT and AST activities of unrestrained control mice compared with vehicle control group. In a state of a generalized catecholamine depletion produced by reserpine, the elevation of ALT (Fig. 4, A) or AST (Fig. 4, B) activities induced by restraint were significantly less than control animals (*p*<0.05). After blockade of *α*-1 or *α*-2 adrenoceptor by prazosin or yohimbine, the magnitude of the increase in ALT and AST activities induced by restraint had a similarly predominant drop compared with control animals (*p*<0.05), which were similar to the effects of reserpine. However, no changes were observed in ALT and AST activities induced by restraint stress after β-1 or β -2 adrenergic receptor antagonist treatment ( betaxolol or ICI 118,551). Regardless of drug treatment, ALT and AST activities of restrained groups were significantly higher than corresponding control groups (*p*<0.05 or *p*<0.01).

**Figure 5:**
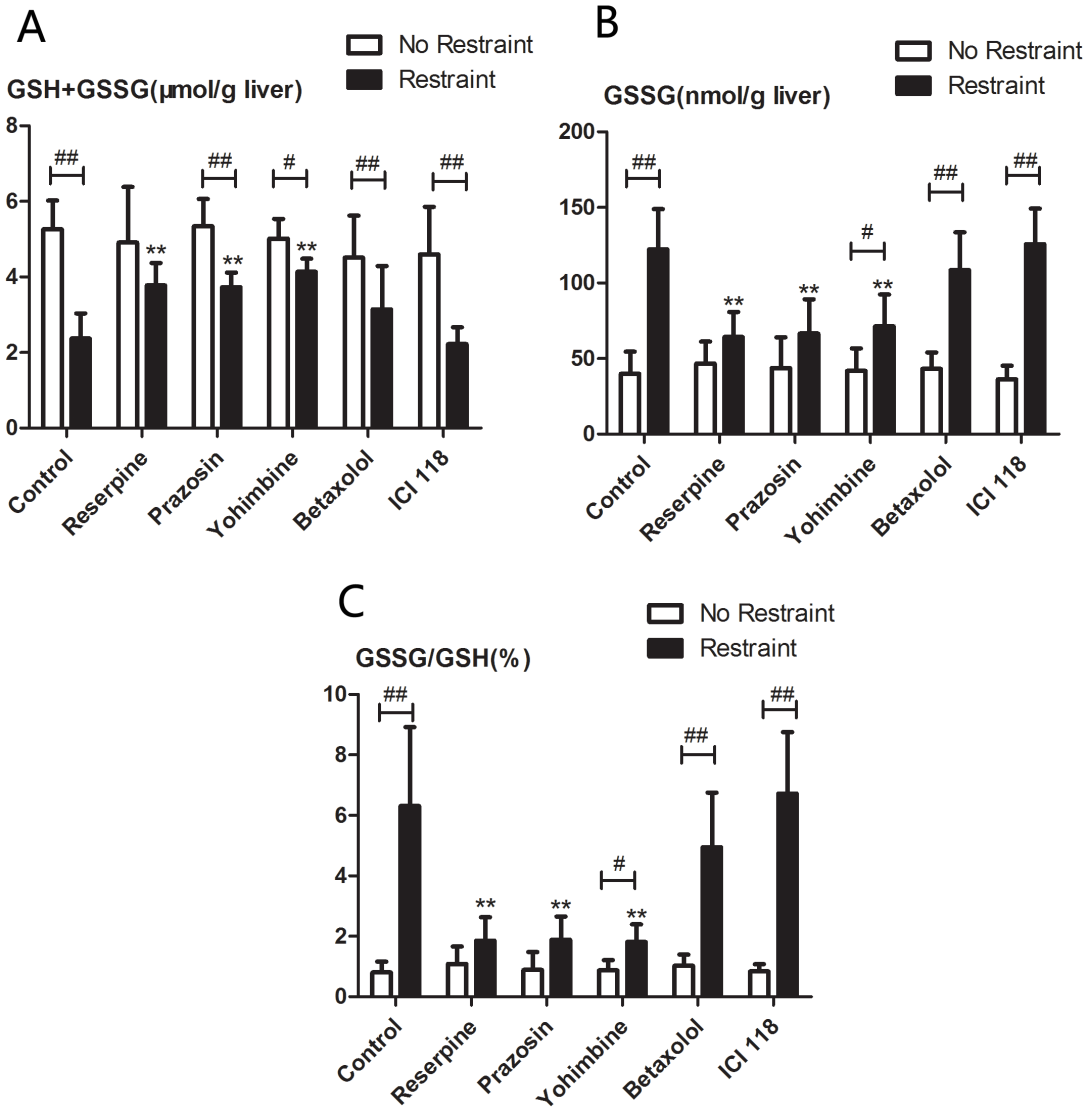
Changes in hepatic glutathione content after pharmacologic manipulation of catecholamine levels and adrenergic receptors in mice followed by exposure to 3 h of restraint. Unrestrained animals were maintained in their cages. Mice were treated by vehicle control, reserpine (2 mg/kg), prazosin (1 mg/kg), yohimbine (2 mg/kg), betaxolol (20 mg/kg) or ICI 118,551 (5 mg/kg) before restraint. Drug-treated animals with 3 h of restraint or unrestraint were sacrificed at the same time points and hepatic total glutathione (GSH+GSSG) (A), GSSG (B) and the GSSG: GSH ratio (C) were measured. Results are expressed as means±SD. There are 5 animals per group. * *p*<0.01compared with control animals (untreated restraint animals); ^#^ *p*<0.05,^ ##^ *p*<0.01 compared with control animals (corresponding unrestrained animals).

As shown in Fig 5, treatment with reserpine, prazosin, yohimbine, betaxolol and ICI 118,551 did not affect hepatic GSH (GSH+GSSG) (Fig. 5, A), GSSG (Fig. 5, B) and GSH/GSSG (Fig. 5, C) ratio of unrestrained control mice compared with vehicle control group. After catecholamine depletion by reserpine or blockade of *α*-1or *α*-2 adrenoceptor by prazosin or yohimbine, the magnitude of the increase in GSSG content and GSH/GSSG ratio and the decrease in GSH content induced by restraint were statistically attenuated compared with that of control animals (*p*<0.01), and these drugs had the same attenuating effect. However, hepatic total GSH, GSSG and GSH/GSSG ratio of restrained animals treated with drugs (reserpine, prazosin, or yohimbine) still significantly different from corresponding control animals (*p*<0.05 or *p*<0.01). Changes of hepatic glutathione content induced by restraint were not affected by beta-1 or beta-2 adrenergic receptor antagonists (betaxolol or ICI 118,551).

### Effect of α-1 and α-2 adrenoceptor antagonists on histological changes in liver of mice subjected to restraint stress

**Figure 6:**
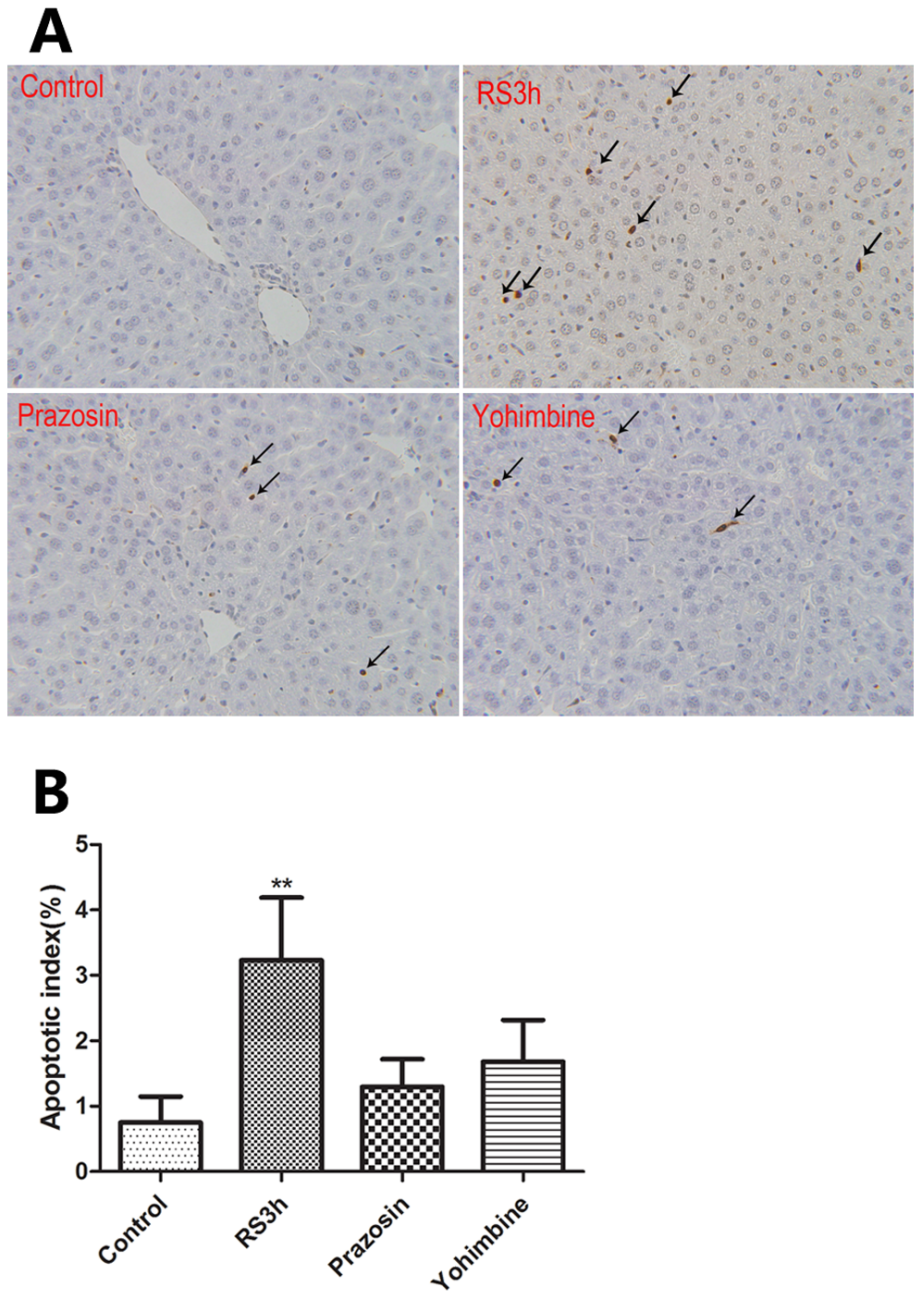
Histological changes (TUNEL assay) in the liver of mice subjected to restrain stress and treatment with prazosin and yohimbine followed by exposure to restraint stress. In the demonstration of apoptosis in situ by the TUNEL assay, TUNEL positive hepatocytes are marked by black arrows (A), and are expressed as apoptotic index (B), ** *p*<0.01 compared with control animals. RS: restraint stress. Magnification×400.

TUNEL assay was used to evaluate the effects of prazosin and yohimbine on hepatocellular apoptosis. After restraint stress for 3 h, apoptotic hepatocytes were frequently observed (Fig. 6, A), while no apparent apoptotic cell was found in the liver tissue of unrestrained mice. Quantitative analysis of TUNEL positive hepatocytes showed that the number of apoptotic hepatocytes in mice exposed to 3 h restraint was significantly higher than that in unrestrained control mice (*p*<0.01) (Fig. 6, B). Treatment with prazosin and yohimbine reduced the number of apoptotic cells in the liver and the two agents exhibited a similar inhibiting effect on the hepatocellular apoptosis.

### Effect of α-1 and α-2 adrenoceptor antagonists on hepatocellular caspase activation and Bax/Bcl-2 expression induced by restraint

**Figure 7:**
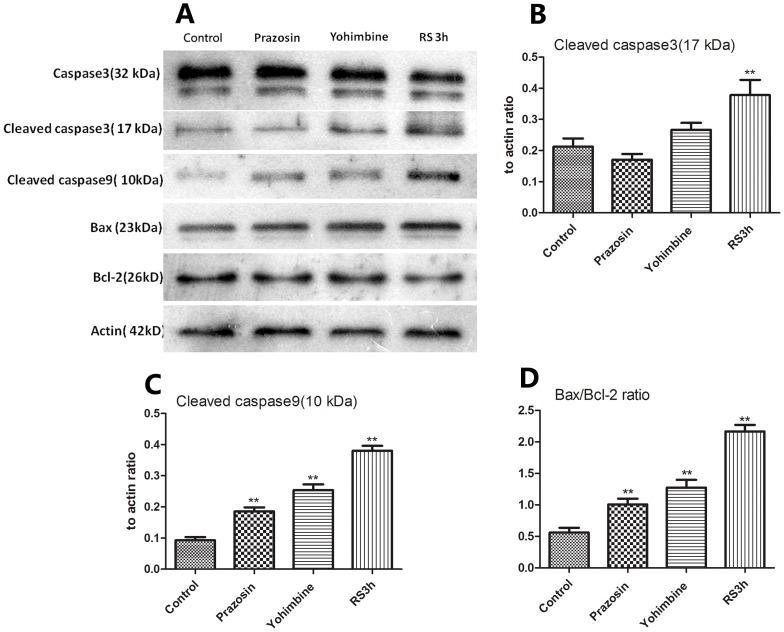
Western blot showing the effect of prazosin and yohimbine on restraint-induced protein expressions of caspase-3, -9, Bcl-2, and Bax in the liver. Samples were obtained from mice subjected to restraint (3 h- duration) and treatment with prazosin and yohimbine followed by exposure to restraint stress. To ascertain the comparison is based on equal protein loading, these samples were also probed with anti-actin antibody. Each lane represents a sample from an individual animal (A).The bands were quantified by densitometry and normalized to actin intensity, and the relative expressions of cleaved caspase 3 (B), 9 (C), and Bax/Bcl-2 ratio (D) are shown, respectively. Data are representative of two independent experiments with similar results. ** *p*<0.01 compared with control animals. RS: restraint stress.

Given the above evidence that *α*1- and *α* 2-adrenoceptor antagonists attenuated hepatocellular apoptosis induced by restraint stress, we investigated whether prazosin and yohimbine could affect caspase-3 activation, which is the key event in apoptotic cell death. As shown in Fig. 7, A, a high level of cleaved form of caspase-3(17 kDa subunit) was detected in the liver of mice subjected to 3 h of restraint, which was much higher than that of control mice (p<0.01) (Fig. 7, B). Pretreatment with prazosin and yohimbine could obviously decrease the level of activated form of caspase-3 induced by restraint.

To explore the mechanism of inhibition of restraint-induced hepatocyte apoptosis by prazosin and yohimbine, we analysed the expression of several molecules related to restraint-induced apoptotic pathways. As shown in Fig. 7, A, restraint stress obviously increased the expression of the proapoptotic factor Bax and the activated form of caspase 9 (Fig. 7, C), and decreased the expression of the antiapoptotic factors Bcl-2 when compared with control group, leading to a high Bax/Bcl-2 ratio (Fig. 7, D). In the prazosin and yohimbine treatment group, the expressions of the above factors induced by restraint were attenuated, but these factors were still unable to return to normal levels of the unrestraint group. These results suggested that prazosin and yohimbine inhibited the hepatocyte apoptosis induced by restraint through affecting the expression of apoptosis-related factors.

## Discussion

Findings of the present study demonstrate an important unfavorable effect of restraint stress on the liver and involvement of catecholamines as well as adrenoceptors in the regulation of the stress. Our results, in agreement with those in many previous studies in mice [27], [28], [29], showed that acute restraint stress applied to mice resulted in elevated serum transaminase levels in a time-dependent manner within 3 h of restraint, although the elevated serum transaminase levels began to decline thereafter. As shown in Fig 1, increase in plasma ALT and AST activity was statistically significant after 1.5 h of restraint, and the activity of the transaminases, an indicator of liver injury according to “Hy's Law”, reached or exceeded 3 times the normal values after 3 h of restraint. Therefore, the animals were experienced 3 h of restraint in the following experiments based on the transaminases results. However, judging by the extent of the rise in serum transaminase activity and changes of hepatic histopathology in restrained mice, restraint stress induced liver injury was indeed mild, which was in agreement with previous report [27].

It has been shown that various types of stress are associated with enhanced free radical generation, a cause of oxidative stress, particularly in the liver [13], [30]. Moreover, oxidative stress has recently been shown to play an important role in various liver diseases[31]. The exacerbation in oxidative stress can be the result of not only an increase in reactive oxygen species (ROS), but also a decrease in antioxidant capacity. Glutathione (GSH) is an important tripeptide thiol antioxidant and its intracellular concentration is an indicator of oxidative stress. Within the cells, GSH exists in two different forms: the reduced (GSH) and oxidized (GSSG) forms. Oxidative stress has a profound effect on the cellular thiol balance and can lead to an increase in the GSSG: GSH ratios in the liver [32]. Therefore, measurement both GSH and GSSG is crucial for understanding of the oxidation-reduction status of cells or tissues. In the present study, restraint stress induced a decrease in hepatic GSH content accompanied by increased GSSG content and GSH/GSSG ratio in a time-dependent manner, further confirming the association of stress with oxidative damage.

Some studies have shown that different forms of stress are able to produce inflammatory responses in different organs [33], [34], which implies that some inflammatory cells and immunomodulatory cytokines, which are usually involved in inflammatory responses, may mediate restraint stress induced liver damage, so we assessed the activity of Kupffer cells (liver-specific macrophages, KCs) by F4/80 immunohistochemistry, and measured the serum levels of IL-6 and TNF-α by ELISA in the process of restraint stress-induced liver injury (data not shown). No significant differences were noted in the density of F4/80-positive Kupffer cells between control and restrained mice. In agreement with previous studies [35], [36], IL-6 and TNF-α levels showed different profiles. IL-6 level was statistically significantly increased after exposure to3 h restraint stress, while no increase in TNF-α was found following restraint. Elevation of IL-6 activity is considered to contribute to the maintenance of homeostasis [36]. These results support the hypothesis that immunomodulatory cytokines may not mediate this kind of stress induced liver injury.

Given the evidence that restraint stress induces liver damage through disrupting the balance of anti-oxidant status, we tested the hypothesis that restraint stress would lead to elevation of hepatic apoptosis. Apoptosis was detected by the TUNEL assay and active caspase-3 was determined by western blotting. The TUNEL-positive nuclei with condensation appearance are considered to be the late stage of apoptosis. Caspase-3 is one of the downstream effector caspases, whose activation is characteristic for caspase-dependent signaling pathways of apoptosis. The present study demonstrated that the level of active caspase-3 was elevated, and TUNEL-positive cells were clearly observed and increased in the liver tissue of mice subjected to 3 h of restraint. These results are in agreement with previous findings that various types of stress can lead to histological changes such as vacuolization, apoptosis, and even necrosis [11], [37], [38].

There is increasing evidence that oxidative stress can induce apoptosis via several pathways. Among them, mitochondria-mediated pathway, including the Bcl-2 family, is the best characterized and believed to be critical in regulating apoptosis [39]. To elucidate how restraint stress regulates apoptotic pathway, we analyzed the levels of activated form of caspases-9, Bax and Bcl-2 by western blotting. Bcl-2 family proteins make a life-or-death decision on the mitochondria. Bax is considered as a proapoptotic and Bcl-2 as an antiapoptotic factor. The ratio of proapoptotic to antiapoptotic proteins (e.g., Bax/Bcl-2) regulates myonuclei integrity and cell survival by controlling mitochondrial membrane permeability [40]. Decreased mitochondrial membrane stability and pore formation initiates the release of cytochrome c, formation of the apoptosome and subsequent activation of caspase-9 and caspase-3 [41]. Our study showed that restraint stress reduced upstream proapoptotic signaling in the Bcl-2 family by increasing the expression of caspase-9 and Bax, and decreasing anti-apoptotic Bcl-2 expression, thus increasing the Bax/Bcl-2 ratio. To our knowledge, here is the first data to indicate that restraint stress induces hepatocyte apoptosis through acting on caspase-9 and Bcl-2 family of apoptotic regulatory proteins.

The mechanism of restraint-induced liver injury is not fully understood. It is well known that the major component of the stress response is the activation of the HPA axis as well as the SAM system, which result in the release of key peripheral mediators glucocorticoids and catecholamines. Previous studies have shown that stress-induced activation of NE release can facilitate activation of the HPA axis in response to acute stress, and α-1 receptor antagonist can significantly attenuate the ACTH response, whereas combined β-1/β-2 receptor blockade has only a modest effect. [42]. In addition, catecholamines can activate the HPA axis by increasing CRF and ACTH release [43], [44]. Several studies have also shown that pharmacological blockade of α or β receptors can inhibit the pathological hyperfunction of the HPA axis during chronic stress [20], which implies that the α or β blockade may affect the stimulation of SAM and HPA axes. Moreover, some studies have ever shown that adrenaline promotes, in a concentration-dependent manner via the activation of α-1B adrenoceptors, hydroxyl radical production in rat hepatocytes [45]. Treatment of rats with noradrenaline stimulates H_2_0_2_ generation in liver mitochondria through the α-2 adrenergic system, but not β adrenergic receptor pathway [46]. These reports confirm a critical role of catecholamines in the stress response. In the present study, we have explored the relationship between inhibition of the SAM system and stress-induced liver injury. Generalized catecholamine depletion with reserpine reduced restraint-induced liver damage, as shown by decreased serum ALT or AST levels and normalization of hepatic glutathione content with improved antioxidant status. Pharmacological blockade of α-1 or α-2 receptors not only led to protective effect on the liver, but also demonstrated attenuation of hepatic necrosis through mitochondria-mediated pathway as evidenced by reduction in expression of antiapoptotic protein Bcl-2, cleaved forms of caspase-3 and 9, and Bax/Bcl-2 ratio. However, treatment with β-1 or β-2 antagonist showed no effects on stress-induced liver impairment. These findings support the hypothesis that catecholamines are released into the circulation and reach the liver during restraint stress, where they bring about the oxidative changes, and activate α-1 and α-2 adrenoceptors, leading to caspase-9 and Bcl-2 family related hepatocytes apoptosis.

In summary, our data demonstrate that restraint stress can increase serum transaminase levels, disrupt the balance between oxidants and antioxidants in the liver and promote hepatocytes apoptosis. In addition, we find for the first time that catecholamines are responsible for restraint-induced liver injury, and α-1 and α-2 adrenoceptors mediate the hepatocyte apoptosis induced by restraint through caspase-9 and Bcl-2 family of apoptotic regulatory proteins. Future studies should be undertaken to investigate the effect of stress on subjects with hepatic disease, for a better understanding of the impact of stress on the liver will help deal with issues of the kind in clinical settings.
